# Preparation and characterization of gamma oryzanol loaded zein nanoparticles and its improved stability

**DOI:** 10.1002/fsn3.1973

**Published:** 2020-12-30

**Authors:** Ubonphan Rodsuwan, Usaraphan Pithanthanakul, Krittiya Thisayakorn, Dudsadee Uttapap, Korawinvich Boonpisuttinant, Savitri Vatanyoopaisarn, Benjawan Thumthanaruk, Vilai Rungsardthong

**Affiliations:** ^1^ Department of Agro‐Industrial, Food and Environmental Technology Faculty of Applied Science Food and Agro‐Industrial Research Center King Mongkut’s University of Technology North Bangkok Bangkok Thailand; ^2^ Expert Center of Innovative Herbal Products (InnoHerb) Thailand Institute of Scientific and Technological Research (TISTR) Pathum Thani Thailand; ^3^ Division of Biochemical Technology School of Bioresources and Technology King Mongkut’s University of Technology Thonburi Bangkok Thailand; ^4^ Department of Thai Traditional Medicine College Rajamangala University of Technology Prathumthani Thailand

**Keywords:** dispersion, gamma oryzanol, nano‐encapsulation, nanoparticles, zein protein, zeta potential

## Abstract

Gamma oryzanol (GO), a bioactive ingredient found in rice bran oil, performs a variety of biological effects such as antioxidant activity, reduction of total cholesterol, anti‐inflammation, and antidiabetes. However, GO is water‐insoluble and normally degrades through oxidation. Thus a nano‐encapsulation technique was investigated to improve its stability and quality. In this research, gamma oryzanol was successfully encapsulated into zein nanoparticles. The fabrication parameters including pH, zein concentration (0.3, 0.4, and 0.5% w/v), and % GO loading (30, 40, and 50% by weight) were investigated. Particle size, zeta potential, yield, encapsulation efficiency and the stability or GO retention during the storage were determined. The morphology of gamma oryzanol loaded zein nanoparticles (GOZNs) was observed by scanning electron micrographs and transmission electron microscope. The increase of zein concentration and % GO loading resulted to an increase of yield, encapsulation efficiency, and particle size. The particle size of the GOZNs ranged from 93.24–350.93, and 144.13–833.27, and 145.27–993.13 nm for each zein concentration with 3 loading levels, respectively. Nano‐encapsulation exhibited higher % GO retention compared with nonencapsulated GO during 60 days storage both at 4°C and −18°C. In vitro study indicated the sustained release of GO in the simulated gastric fluid followed by simulated intestinal fluid. This finding indicated a high potential for the application of insoluble GO with improved stability by encapsulation with the hydrophobic zein protein.

## Graphical abstract

The bioactive compound and gamma oryzanol (GO) were encapsulated in zein nanoparticles (ZNs) to obtain gamma oryzanol loaded zein nanoparticles (GOZNs), using a liquid‐liquid dispersion. The effect of pH, zein concentration, and % GO loading on the properties of GOZNs showed that the increase of zein concentrations and % GO loading resulted in larger sizes of the nanoparticles as well as the increased % encapsulation efficiency and % yield of GOZNs. The encapsulation results in the higher stability of GO during 60 days storage.

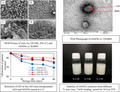

## 1 INTRODUCTION

Rice is a significant food resource and widely cultivated in many parts of the world. Thailand is one of the world leaders for rice exporter. Rice bran and germ, by‐products from the milling process into white rice, also provide beneficial nutrition such as vitamins B, and E, beta‐carotene, anthocyanin, gamma oryzanol (GO), and gamma‐aminobutyric acid (GABA). GO can be extracted as a mixture of ferulic acid (4‐hydroxy‐3‐methoxy cinnamic acid) esters with phytosterols and triterpene alcohols (Kim et al., 2013). The ferulic acid ester in rice is well known for its biological effects, including anti‐inflammation, antidiabetes, antiaging, anticholesterol actions, and relieving menopausal symptoms (Khalid et al., 2017). GO is a potent inhibitor of iron‐driven hydroxyl radical formation and associated with antioxidant activity in stabilizing lipids (Suh et al., 2007). However, GO is a hydrophobic, water‐insoluble compound with low absorption and poor bioavailability (Kim et al., 2013) which limit its application. The enhancement of its solubility would widen the application of GO in food, cosmetics, and related products.

Encapsulation has been proposed as an effective technique for maintaining the quality and stability of active food ingredients (Padua & Wang, 2009) against oxidation and provide better solubility (Raharjo et al., 2019). The small particle size could help the delivery of the bioactive compounds within the body. Nanotechnology indicates a high potential for the development of functional foods, without affecting consumer perception and improving the uptake of individual components. Recently, there are considerable interests in developing high‐performance delivery vehicles to protect bioactive compounds, improve bioactive compound bioavailability for improving drug delivery to the target, and enhancing the stability of active chemicals for drug formulation (Huang et al., 2020). Various techniques were used for the nano‐encapsulation of bioactive compounds such as spray drying and coacervation, including solid‐lipid nanoparticle, and liquid‐liquid dispersion method used for encapsulation of bioactive compounds by zein protein (Zhong & Jin, 2009). For microencapsulation of GO, native rice starch and pregelatinized rice starch were used using spray drying (Moongngarm et al., 2016), while Khalid et al.(2017) used microchannel emulsification with medium‐chain triglyceride oil to encap the GO. The particle sizes obtained ranged around 5–60 µm and 26–28 µm, respectively. However, it is wellknown that reducing the particle size of encapsulated particle to nano‐size would improve the uptake of encapsulated bioactive compound in cells (Luo et al., 2011).

Zein is the prolamine protein from corn endosperm and a significant by‐product in the corn starch industry and generally recognized as safe by the US Food Drug Administration. The current production of corn starch sources in Thailand is estimated to be 2.26 million tons (Win, 2017). Zein has several properties allowing it to self‐assemble into different structures such as film, sponges, and spheres (Wang & Padua, 2010). In the pharmaceutical and food areas, zein shows a high potential for application as a biomaterial for the nano‐encapsulation of bioactive compounds with the ability to provide moisture, an oxygen barrier, and high thermal resistance (Huang et al., 2020). Zein can assist the control release of encapsulated hydrophobic compound such as polyphenols, vitamins, and omega‐three fatty acids into nonaqueous environment and prolong its shelf life (Donsì et al., 2017; Wu et al., 2012).

Though zein nanoparticles have been prepared and characterized for over a decade, to the best of our knowledge, there has been no report on the use of zein for nano‐encapsulation of GO. In addition, using both zein and GO would add value to the abundant by‐products from corn and rice, which contain highly functional ingredients. In present study, GO loaded zein nanoparticles (GOZNs) were fabricated and characterized in order to improve GO dispersibility and stability during a long storage. The effects of zein concentration, and % GO loading on particle size, zeta potential, encapsulation yield, and efficiency were determined. Long‐term storage stability of GO in the nanoparticles was measured during 60 days. In vitro release of GO from the nanoparticles was measured. The nanoparticle structure was also examined.

## 2 MATERIALS AND METHODS

### 2.1 Materials

GO was purchased from Tsuno Rice Fine Chemicals Co., Ltd. (Wakayama, Japan) with purity ≥99%. Zein protein was provided by Sigma‐Aldrich Co., Ltd. (USA). All other chemicals and solvents were analytical grade, while Tween 80 and lecithin were food‐grade, supplied from Union Chemical 1986 Co., Ltd. (Thailand).

### 2.2 Preparation of ZNs: Effect of pH and zein concentration

A liquid‐liquid dispersion was modified from the method used by Luo et al., (2013). The effect of the surfactant ratio between tween 80 and lecithin on the particle size and zeta potential of the particles obtained was studied (data not shown), and the ratio at 1:2 (w/w) was selected. pH stability of the zein suspension was studied. The surfactant was dissolved at 0.025% (w/v) in 0.1 M citrate solution, and the pH was adjusted to pH 5, 7, 7.4, 8, and 9 to evaluate the pH stability of the ZN dispersion. Twenty ml of 0.4% zein solution (w/v) in 85% ethanol was mixed with the surfactant solution (60 ml) to prepare 0.1% (w/v) zein dispersion at a various pH level and stirred using a homogenizer (Ultra‐Turrax T25, IKA, Germany) operated at 15,000 rpm for 10 min in an ice bath. The ethanol in the dispersion was removed using a vacuum rotary evaporator and centrifuged. The particle size and zeta potential of freshly prepared zein nanoparticles (ZNs) and those of 24 hr storage at 25°C ± 2°C were measured.

In order to increase the yield of ZNs and GOZNs, the zein concentration in the dispersion was increased from 0.1% to 0.25, 0.5, 0.6, and 0.7% (w/v) with pH 8 following the above method. The dispersions were stored at room temperature (25°C ± 1°C) for 24 hr and observed for their stability.

### 2.3 Preparation of GOZN with different zein concentrations and % GO loading

To study the effect of zein concentrations and % GO loading on the properties of the nanoparticles obtained, GOZNs were fabricated as follows: GO was dissolved in 1% isopropanol (w/v) at room temperature and added dropwise into 20 ml of zein solution in ethanol using a magnetic stirrer at 450 rpm for 10 min. Then, the sample was added to 60 ml of 0.1 M citrate solution containing the surfactant (tween 80/lecithin at 0.025%, w/v), and the final concentration of zein dispersion was 0.3, 0.4, and 0.5% (w/v), respectively (with pH of the aqueous phase between 8 and 9). The mixture was homogenized and evaporated as described above. Three % levels of GO loading at 30, 40, and 50% of zein (w/w) were studied. Each condition was expressed as % zein concentrations/ % GO loading (0.3/30, 0.3/40, 0.3/50, 0.4/30, 0.4/40, 0.4/50, 0.5/30, 0.5/40, and 0.5/50).

### 2.4 Particle size and Zeta potential

Particle size and distribution of the nanoparticles, as well as polydispersity index (PDI), were measured by dynamic light scattering (DLS) using a DelsaTM nanoparticle analyzer (Beckman Coulter, Fullerton, CA). Surface charges of different samples were measured using a Laser Doppler Velocimeter (Zetasizer Nano ZS90, Malvern, UK) with a folding capillary cuvette. Zeta potential of the samples was converted from the measured electrophoretic mobility.

### 2.5 Colloidal stability of ZNs and GOZNs

After preparation for GOZNs, 10 ml of each dispersion was poured into a glass bottle and sealed with a plastic cap and stored overnight at 25°C ± 1°C. The stability of the dispersion prepared at different pH and from different zein concentration was observed for changes in particle size distribution which could result to phase separation after 24 hr.

### 2.6 Encapsulation efficiency and yield

The encapsulation efficiency (EE) of GO in GOZNs was defined as the GO content encapsulated in ZNs following Sakulkhu et al., (2007). Five mg of GOZNs was dispersed in 4 ml of a mixture of methanol and dichloromethane (1:1 v/v), sonicated for 10 min, and filtered before the injection. The GO content in the filtrate was measured using high‐performance liquid chromatography, HPLC (Agilent Technology 1,200 series, Germany). The HPLC system was attached to HPLC 1,200 series UVVIS LC Detector equipped with an Eclipse Zorbax XDB‐C 18 (Agilent, 250 × 4.6 mm, 5 µm) analytical column and a Zorbax XDB‐C18 (Agilent, 12.5 × 4.6 mm, 5 µm) guard column. The mobile phase consisted of a mixture of methanol, acetonitrile, dichloromethane, and acetic acid at a ratio of 55:35: 9.5:0.5. Percentages of encapsulation efficiency and yield were calculated as follows:

% Encapsulation efficiency = (Total GO amount in nanoparticles/ Total GO amount added to the preparation of the nanoparticles) × 100 (1)

% Yield = (A/B) × 100 (2)

where A = Dry weight of nanoparticles.

B = Total initial weight of the zein and GO used for the nanoparticle preparation.

### 2.7 SEM, TEM, and FTIR

Image study on morphological structures of the samples was carried out using a scanning electron microscope, *SEM* (JSM‐6610LV, JEOL, Japan), at a voltage of 5 kV. GOZNs from 0.3/30 were separated by centrifugation and dried using a freeze drier (Alpha 1–4 LSCplus, Christ, Germany). The samples were mounted on specimen stubs and coated with a thin (<20 nm) conductive platinum layer using a sputter coater. For TEM (Transmission Electron Microscope), the sample was stained with 1% (v/v) uranyl acetate (Sigma‐Aldrich, St. Louis, Mo, USA) for 30 s, dried and observed via JEM‐1400 Electron Microscope, JEOL (Japan). Fourier transform infrared spectroscopy (FTIR) spectra of zein, GO, ZNs, and GOZNs were determined following Luo et al. (2011). The samples were mixed with potassium bromide to form a thin film at 4.5 Bar for 8 min and subjected to FTIR 4,100 (Jasco, Japan).

### 2.8 Long‐term storage ability of GO

The dried GOZNs from 0.3/30 (particle size 127 nm) and free GO were sealed in the glass vial (20 ml) and stored at 4°C and −18°C in the refrigerator for 60 days. The samples were taken out every 15 days to determine for the retention amount of GO by HPLC following the methods described above.

### 2.9 In vitro release of GO from GOZNs and free GO

The GO release profile of GOZNs and remaining free GO in SGI tract containing enzymes was evaluated following Luo et al. (2011). In vitro release was studied with 10 mg of GOZNs and remaining free GO in simulated gastric fluid (SGF) for 0.5 hr followed by simulated intestinal fluid (SIF) for 6 hr. The samples were first mixed in 30 ml SGF pH 1.2 with 0.1% pepsin (w/v) and incubated in a shaking water bath (37 °C, 100 rpm). The digestion was stopped by raising pH to 7.5 (1 M NaOH) and centrifuged (9,000 *g*, 50 min) to separate the supernatant. The amount of GO from GOZNs released, and remaining free GO was determined by HPLC. Then, 30 ml SIF pH 6.8 with 1% pancreatin (w/v) was added to the precipitate and incubated for 6 hr (37 °C, 100 rpm). The GO release medium and remaining free GO (5 ml) were collected and analyzed for GO content at predetermined times (1, 2, 4, and 6 hr).

### 2.10 Statistical analysis

The result is shown as the mean ± standard deviation (*SD*). Analysis of variance (ANOVA) was used to analyze the statistical significance using SPSS 22.0 for Windows (SPSS Inc., Chicago, III, USA). The data were subjected to ANOVA, and a comparison of means was carried out by Duncan's Multiple Range Test (DMRT).

## 3 RESULTS AND DISCUSSION

### 3.1 Preparation of ZNs: effect of pH and zein concentration

Table 1 shows that the particle size (118–129 nm) of ZNs prepared at pH 7.4 and 8 was not significantly different after the preparation and also after 30 days storage. However, a minor change of their zeta potential was observed after the storage. An increase of pH to 9 resulted to significantly smaller ZNs at 58.0 ± 2.0 nm which changed to larger particle (87.9 ± 0.9 nm) after 30 days. Protein coagulation occurred rapidly after the mixing at pH 5 and precipitation of the zein particles was observed after some hours with pH 7. Zein protein contains roughly hydrophobic and hydrophilic amino acid residues. Zein was denatured at pH 5 and 7 which closed to its isoelectric point (pH 6.8). Shukla and Cheryan (2001) reported that zein is rich in glutamic acid (26%) and can be dissolved well in alkaline solution. Deprotonated carboxylic groups in glutamate occurs when pH of the aqueous phase increased to a basic condition, resulting in a negative charge on the zein molecule surface led to smaller zein particles (Zhong & Jin, 2009). The particle size of zein depends on many factors such as zein concentrations, percentage of ethanol‐water solvent, pH of the aqueous solution (Zhong & Jin, 2009), and phase separation based on differential solubility (Podaralla & Perumal, 2012). Under acidic conditions, zein tends to form larger particles and polydispersity than in neutral and base solutions. Zein has been reported to have a monomeric form at pH > pI, and particle size decreased with an increase of pH (Podaralla & Perumal, 2012).

**TABLE 1:** Particle size and zeta potential of zein nanoparticles prepared from 0.1% zein emulsion with different aqueous pH and storage at 4^O^C for 30 days

pH	After preparation		30 days storage	
	Particle size (nm)	Zeta potential (mV)	Particle size (nm)	Zeta potential (mV)
7.4	118.0 ± 8.2^bA^	−31.8 ± 1.5^aB^	116.9 ± 11.9^bA^	−26.0 ± 2.8^aA^
8	129.5 ± 5.0^bA^	−31.9 ± 1.2^aB^	125.7 ± 8.5^bA^	−27.5 ± 0.7^aA^
9	58.0 ± 2.0^aA^	−31.0 ± 3.4^aA^	87.9 ± 0.9^aB^	−31.0 ± 1.3^bA^

Note

Values were expressed as means ± *SD* (*n* = 3). Different superscript letters (a‐e) in the same column for particle size, and zeta potential, and letters (A‐B) in the same row for particle size, and zeta potential mean significantly different (*p* < .05).

**FIGURE 1:**
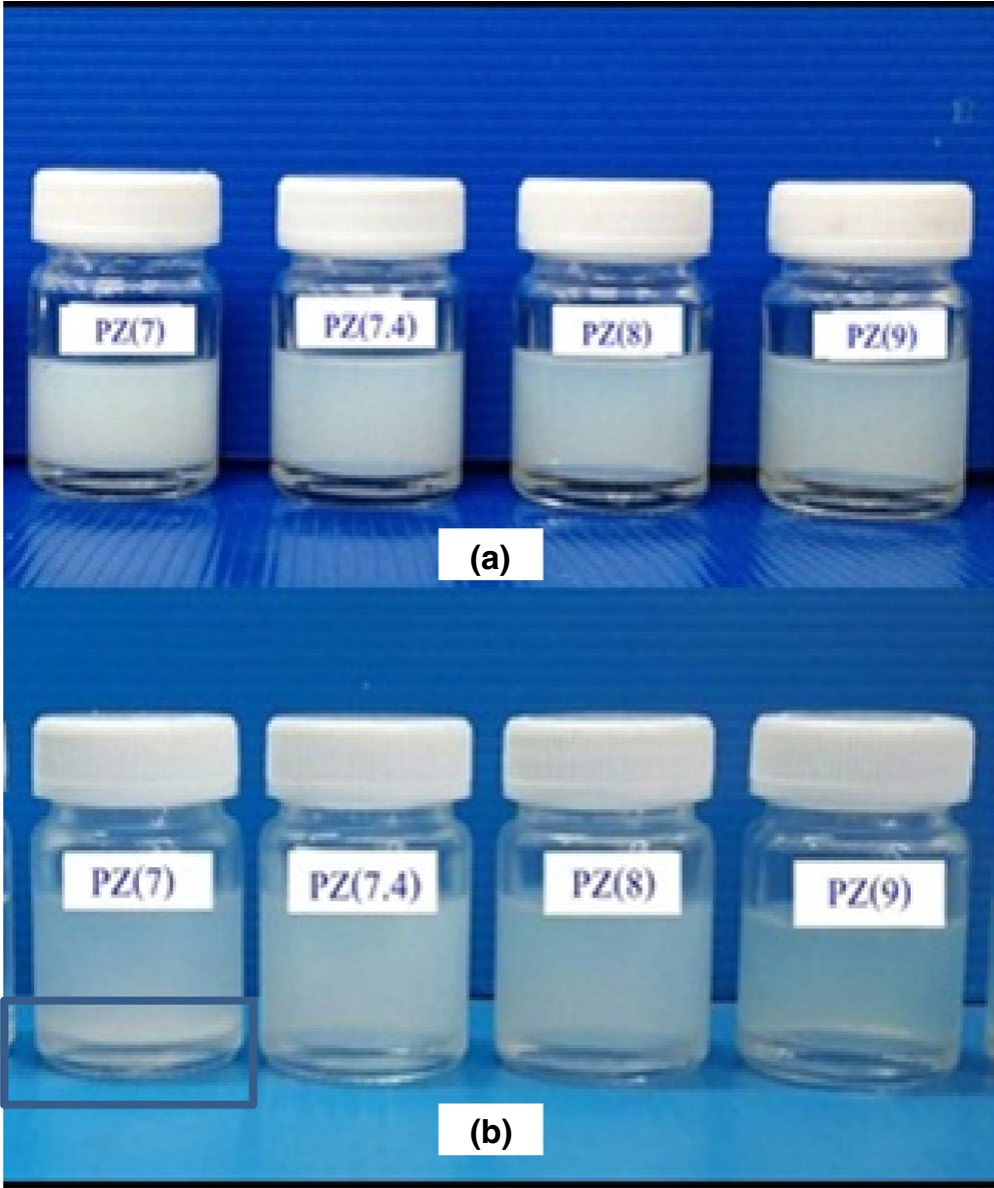
Dispersion of zein nanoparticles, prepared at different pH, after the preparation (a) and 24 hr storage at 25°C (b). Precipitation in the square (pH 7) could be observed

Zeta potential is an essential parameter for understanding the nanoparticle surface and for predicting the stability of the nanoparticles in the dispersion. ZNs dispersion prepared at pH 7.4, 8, and 9 indicated surface charge at around −31 mV which exhibited stable colloid (Kumar & Dixit, 2017). The ZNs prepared at pH 9 presented high degree of colloidal stability and the surface charge did not change even after the storage at 4^O^C for 30 days as indicated in Table 1 and Figure 1.

**FIGURE 2:**
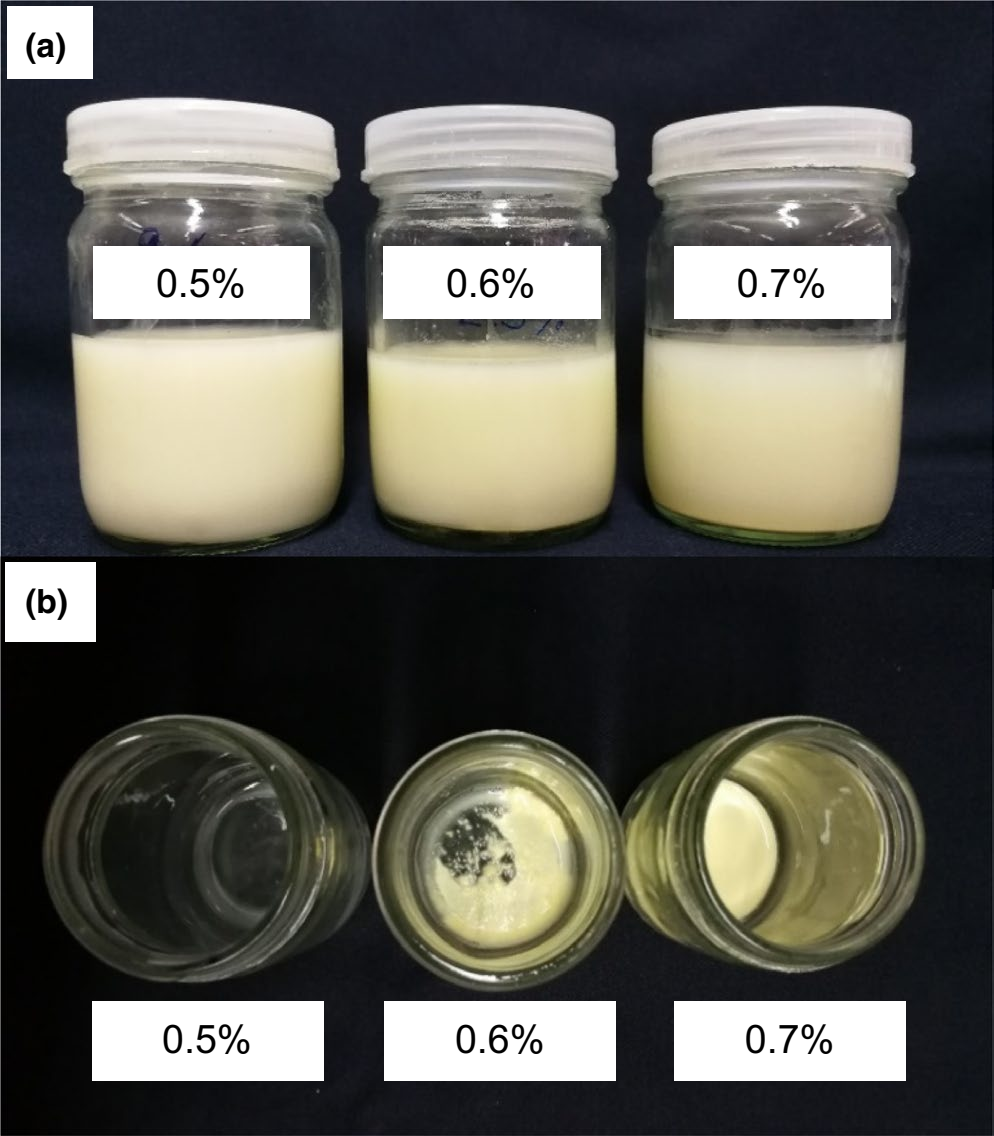
Precipitation of ZNs prepared with different zein concentration (pH 8) after the preparation (a), and 24 hr storage at 25°C (b)

When zein concentration was increased from 0.1% to 0.25, 0.5, 0.6, and 0.7% (w/v), a precipitation (Figure 2) was observed in the ZNs dispersion of zein concentration higher than 0.5% (w/v) thus 0.5% zein concentration was selected for further study. An increase of pH of the aqueous phase from 7.4 to 9 caused similar results that a measurable decrease in the final mean particle size of zein in the dispersion, from 213.80 ± 0.82 nm to 141.40 ± 0.61 nm, which were larger than the particle size obtained with the use of 0.1% zein (w/v) as shown in Table 2. The results were corresponding with the study of Zhong and Jin (2009), who reported the monotonic increase of particle sizes and the viscosity of zein, resulting in a larger size. During liquid‐liquid dispersion, stronger inertia occurred with higher zein concentrations, leading to droplet deformation. Larger droplets may be formed during the dispersion process and, thus resulting to larger particles (Liu et al., 2019; Zhong & Jin, 2009).

**TABLE 2:** Some properties of zein nanoparticles (ZNs) prepared using 0.5% zein concentration (w/v) with different aqueous pH

pH of citrate solution	ZNs size (nm)	Zeta potential (mV)	Polydispersity index (PDI)
7.4	213.80 ± 0.82^b^	−24.33 ± 3.51^b^	0.12 ± 0.01^b^
8	185.10 ± 0.79^b^	−32.50 ± 0.45^a^	0.07 ± 0.03^a^
9	141.40 ± 0.61^a^	−33.43 ± 2.93^a^	0.03 ± 0.01^a^

Note

Values were expressed as means ± *SD* (*n* = 3). Different superscript letters in the same column mean significantly different (*p* < .05).

Abbreviation: ZNs, zein nanoparticles.

The zeta potential of the nanoparticle ranged from −24.33 ± 3.51 to −33.43 ± 02.93. The reduction of zein nanoparticle size was corresponding with the zeta potential and the polydispersity index (PDI). Danaei et al., (2018) reported that PDI values lower than 0.05 were mainly seen with highly monodisperse standards, while PDI values higher than 0.7 indicated an extensive particle size distribution of the sample.

### 3.2 Effect of zein concentrations and % GO loading

#### 3.2.1 Particle size, zeta potential, encapsulation yield and efficiency, and colloidal stability

The increase of zein concentration and % GO loading tended to increase the particle size, encapsulation yield, and efficiency as presented in Table 3. The particles obtained from the use of zein at 0.3% (w/v) and all GO loading 30, 40, and 50%, ranged 127–350 nm while those of 0.4% (w/v) with 30 and 40% loading were 195, and 445 nm, respectively (Table 3). The increase of zein concentrations to 0.5% with all GO tended to yield microparticles (613–993 nm). Reducing the particle size of encapsulated particle to nano‐size would improve the uptake of encapsulated bioactive compound in cells (Luo et al., 2011). The encapsulation efficiency ranged from 61.46%–77.97%, 67.16%–71.90%, and 60.32%–90.99% for each zein concentrations (0.3, 0.4, and 0.5% w/v) with three % GO loadings, respectively, while the highest yield of nanoparticles, at 75.03%, was obtained with 0.5% zein concentrations at 50% GO loading. Similar phenomena were reported with the encapsulation of α‐tocopherol by zein protein, where the encapsulation efficiency increased with the increase of zein protein (Luo et al., 2011).

**TABLE 3:** Some properties of GOZNs prepared using different zein concentration/ % GO loading

% Zein conc/% GO loading	Particle size (nm)	Zeta potential (mV)	Polydispersity index (PDI)	Yield (%)	Encapsulation efficiency (%)
0.3/0	111.24 ± 0.78^a^	−35.17 ± 2.46^h^	0.06 ± 0.02^a^	‐	‐
0.3/30	127.87 ± 1.32 ^a^	−57.16 ± 5.83 ^b^	0.08 ± 0.01 ^a^	36.65 ± 1.47	61.46 ± 3.32^a^
0.3/40	145.00 ± 0.35^a^	−42.80 ± 2.28^ef^	0.09 ± 0.01^a^	43.28 ± 1.65	59.66 ± 4.23^a^
0.3/50	350.93 ± 33.60 ^b^	−39.30 ± 0.52^fgh^	0.39 ± 0.03^bcd^	67.92 ± 1.94	77.97 ± 6.51^d^
0.4/0	144.13 ± 0.95^a^	−37.60 ± 3.05^h^	0.06 ± 0.01^a^	‐	‐
0.4/30	195.17 ± 2.89^a^	−37.20 ± 1.74^gh^	0.20 ± 0.03^ab^	41.78 ± 1.69	67.16 ± 6.29^b^
0.4/40	445.03 ± 12.38^b^	−54.13 ± 3.93^bc^	0.48 ± 0.01^cd^	54.95 ± 0.76	70.87 ± 8.57^c^
0.4/50	833.27 ± 113.62^d^	−40.96 ± 0.83^fg^	0.52 ± 0.02^d^	69.21 ± 1.53	71.90 ± 6.19^cd^
0.5/0	185.10 ± 0.79^b^	−32.50 ± 0.45^h^	0.07 ± 0.03^a^	‐	‐
0.5/30	613.07 ± 11.12^c^	−47.33 ± 0.95^de^	0.27 ± 0.04^abc^	54.52 ± 2.04	60.32 ± 6.70 ^a^
0.5/40	846.27 ± 52.63 ^d^	−65.36 ± 0.47 ^a^	0.46 ± 0.12^cd^	67.23 ± 0.76	76.25 ± 8.34^d^
0.5/50	993.13 ± 122.71^e^	−54.76 ± 4.53^bc^	0.65 ± 0.16 ^cd^	75.03 ± 2.08	90.99 ± 4.01^e^

Note

Values were expressed as means ± *SD* (*n* = 3). Different superscript letters in the same column mean significantly different (*p* < .05).

Abbreviations: GOZNs: gamma oryzanol encapsulated zein nanoparticles, GO: gamma oryzanol.

**FIGURE 3:**
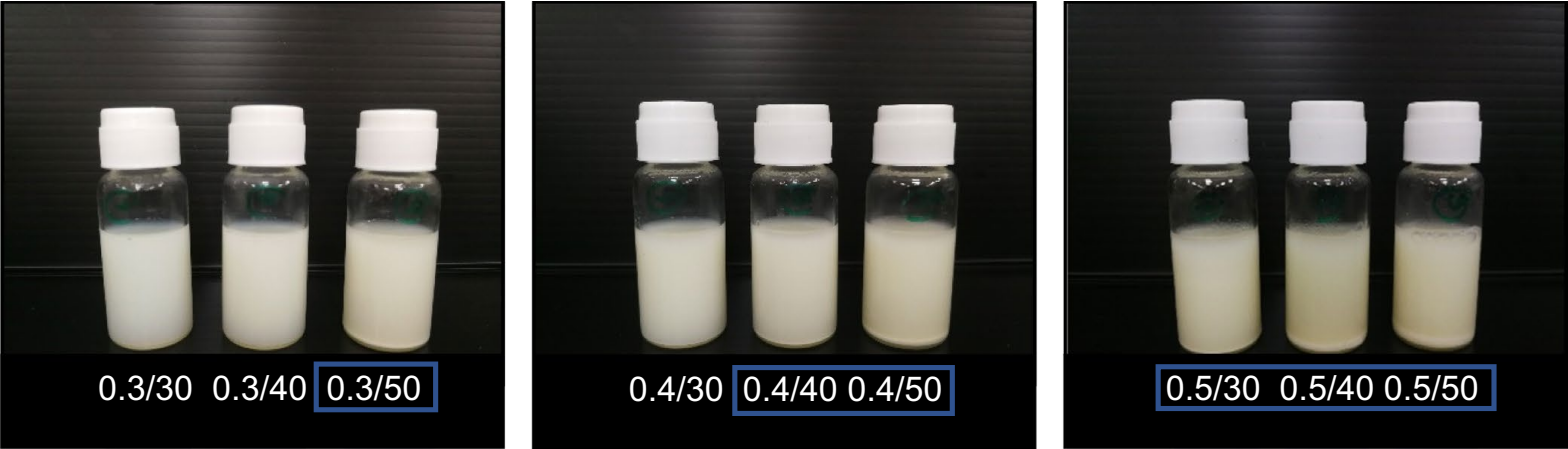
GOZNs emulsions prepared using different zein concentration/ % GO loading, stored for 24 hr at 25°C. Precipitation in the square could be observed

The poor aqueous solubility of GO often limits its application in functional food and beverage (Kim et al., 2013). The encapsulation of GO in the zein nanoparticle will help the dispersion of this bioactive compound in a food system (Padua & Wang, 2009; Raharjo et al., 2019). Determining the colloidal stability of the micro/nano‐dispersion is necessary since food processing is usually a complex procedure. Stable dispersion with no precipitation was observed with the nanoparticles obtained from 0.3/30, 0.3/40, and 0.4/30, their GOZNs were as small as 127.87, and 145.00, and 144.13 nm, with PDI 0.08–0.20 as shown in Figure 3. In comparison, precipitation after 24 hr was observed with the zein particles larger than 350 nm (PDI 0.27–0.65). Zein can be aggregated easily, and it has been reported that zein has an interparticle hydrophobic attraction with a formation of a thin layer of particles in the bottom flask during storage (Donsì et al., 2017).

An increase of zeta potential was observed in most experiments with the increase of zein concentration and % GO loading. The anionic surfactant, lecithin, increased the electrostatic interactions between zein particle and enhance the surface charge of the dispersion. Eventhough very high surface charges such as −65.36 and −54.76 mV were measured from the dispersion with microparticles (846 and, 993 nm), precipitation occurred at 24 hr storage. System instability resulted when attractive interparticulate forces predominated over repulsive interparticulate forces, which caused an increase in particle size and particle aggregation, respectively (Guo et al., 2008). The results indicated that the size of the particle had more influence on the dispersion stability during the storage than the surface charge shown.

#### 3.2.2 SEM, TEM, and FTIR

**FIGURE 4:**
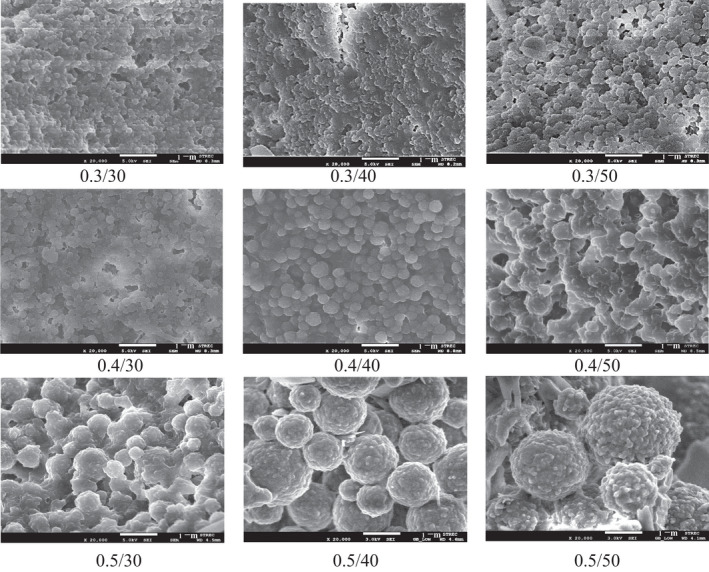
SEM images of GOZNs prepared using different zein concentration and % GO loading at magnification of 20,000 folds

SEM images of the micro/nanoparticles are shown in Figure 4. Most particles are spherical and present the size in accordance with the particle sizes measured in the dispersion (Table 3). However, microparticles larger than 2 µm with textured surface were observed in the samples from 0.5/40 to 0.5/50. This could be the formation of larger self‐assembled micro/ nanosphere of the zein with increased zein concentration (Luo et al., 2011) during the centrifugation and freeze drying.

**FIGURE 5:**
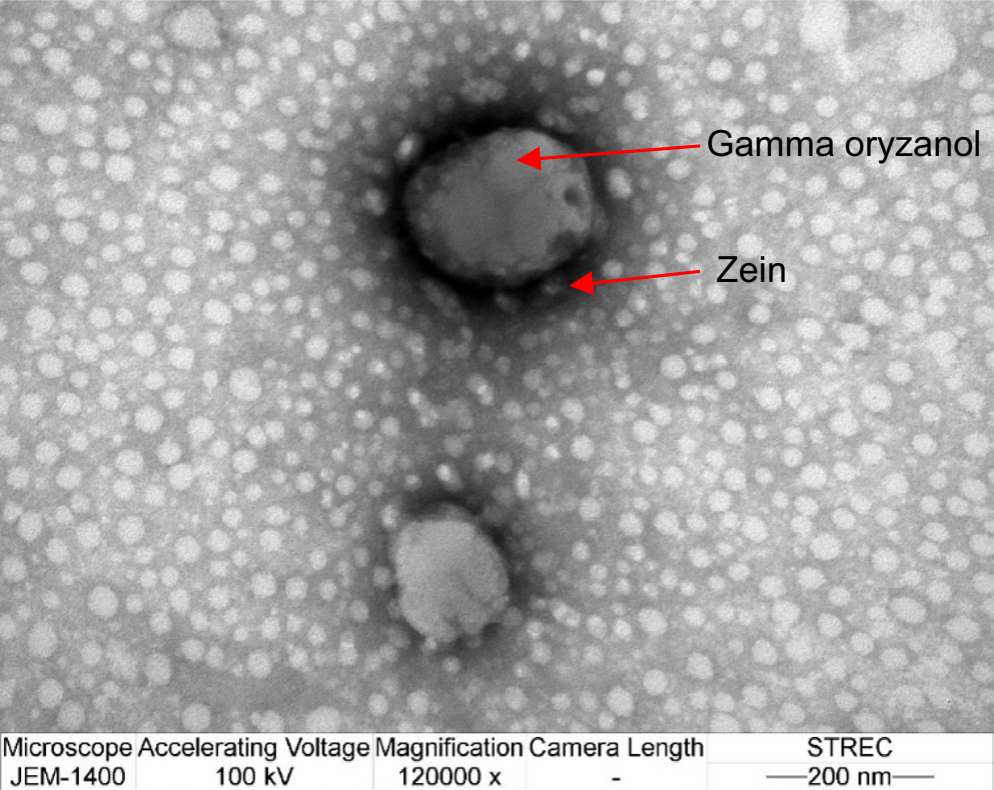
TEM picture of gamma oryzanol encapsulated zein nanoparticle (× 120,000)

The transmission electron microscope (TEM) is a potent tool for characterizing the morphology of encapsulated nanoparticles using a focused beam of electrons that is transmitted. Zein is a protein with negative charge, which can bind to uranyl acetate resulting in black shade. Low‐density material of GO is seen in the core section as a light shade and surrounded by the high density of ZNs in a darker shade. The TEM image indicates that GO was encapsulated by ZNs (Figure 5).

**FIGURE 6:**
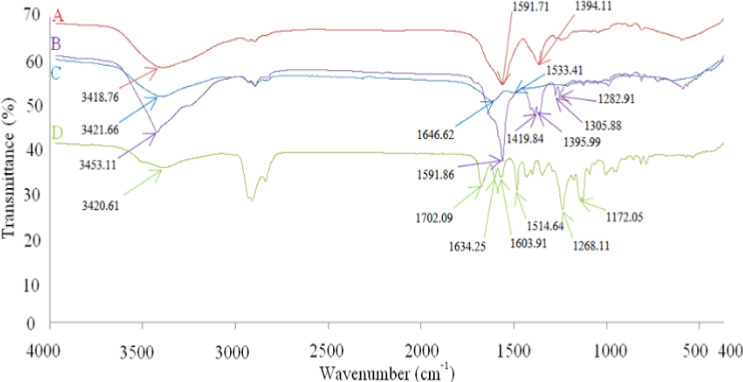
FTIR spectrum of zein nanoparticle (a), gamma oryzanol encapsulated zein nanoparticle (b), zein protein (c), and gamma oryzanol (d)

FTIR has been applied to characterize the interactions of nanoparticles of ZNs, GOZNs, zein protein, and pure GO (Liang et al., 2017). The hydrogen bonds were exhibited at wavelengths of 3,418.76, 3,453.11, 3,421.66, and 3,420.61 cm^‐1^, respectively (Figure 6). The hydrogen bonds are formed between the amines of glutamine amino acids in proteins and hydroxyl groups (‐OH) in GO (Luo et al., 2011). The hydrogen bond of GO at a spectral of 3,420.61 cm^‐1^ disappeared when GO combined with the zein protein and moved to the 3,453 cm^‐1^ range in GOZNs. The spectrum of zein from 1,646.62 shifted to 1,591.86 cm^‐1^ when ZNs were formed, while at 1,533.41 cm^‐1^ of the Amide II, it disappeared. The semi‐functional group of ferulic acid, which is in the spectra range of 1,634 and 1,603 cm^‐1^ caused by *a trans* double bond conjugated to an aromatic ring. The functional group of GOZNs shifted and disappeared from the spectra at many wavelengths, such as 1,591 cm^‐1^, causing the combination of the carbonyl stretching groups of GO with Amide I of the zein protein. These results implied the encapsulation of by zein nanoparticles.

### 3.3 GO retention during storage

**FIGURE 7:**
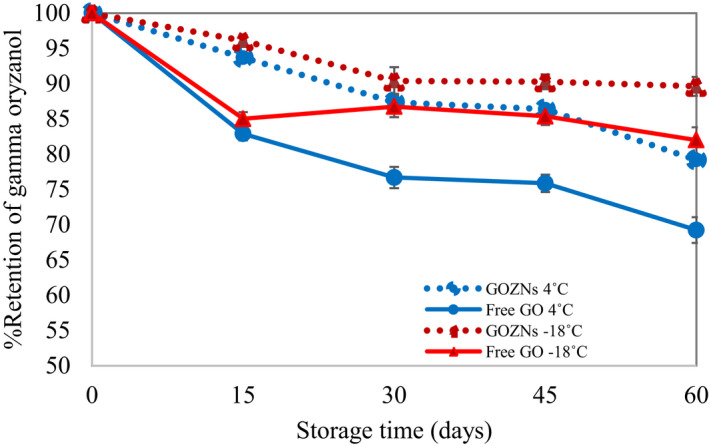
Retention of GO in free GO (nonencapsulated GO) and GOZNs stored at 4°C and −18°C

Generally, GO is susceptible to oxygen in the air and degraded during food processing and storage (Moongngarm et al., 2016). The retention of GO decreased to 87.12 and 79.62% at 30 days and 60 days storage at 4^o^C, which was higher than that of free GO at 83.21, and 69.32%, respectively (Figure 7). The results showed that encapsulation in zein nanoparticles could improve the stability of GO inside. Shin et al. (1997) reported that GO was degraded through oxidation resulting from oxygen in the air and sunlight (Moongngarm et al., 2016) and the loss of GO was around 16.4% and 62.7% after 35 days and one year storage, respectively. Comparing to this, the retention of GO in GOZNs reduced to 90.13, and 90.12 at 30 days, and 60 days storage at −18^o^C while the retention of free GO was 87.53 and 82.32%, respectively. At lower temperatures, degradation of the bioactive compound could be prolonged, and the antioxidant activity could be preserved because of the reduced activity of related enzymes (Qiu et al., 2014). The results confirmed that the nano‐encapsulation could delay the oxidation of GO by protecting the core material from the environment.

### 3.4 In vitro release of GO from GOZNs and free GO

**FIGURE 8:**
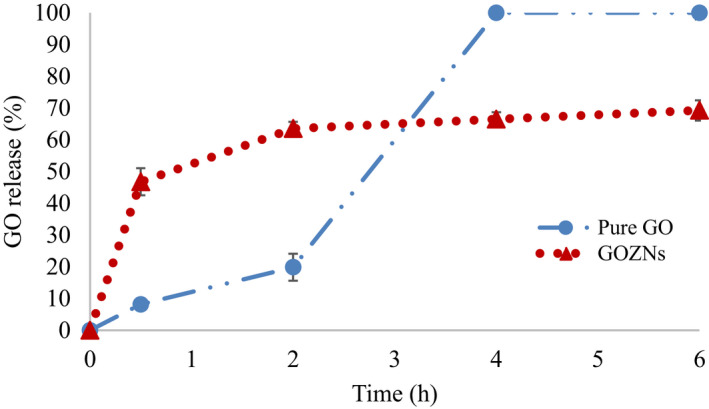
In vitro release profile of GOZNs and remaining free GO in simulated gastric fluid (SGF) for 0.5 hr followed by simulated intestinal fluid (SIF) for 6 hr

The release profile of GOZNs and remaining free GO over time in a simulated gastrointestinal fluid under with pepsin and pancreatin at 37°C for 6 hr are expressed in Figure 8. Both studies were characterized by different two‐step phases. The high GO release of GOZNs in SGF 30 min, at 46.79% was mainly due to the collapse of a sample from zein enzymatic breakdown (Luo et al., 2011). Adding of SIF at 0.5 hr and incubated further showed that the GO release was slowed down to 63.57% at 2 hr and 69.26% at the end of incubation for 6 hr. Comparing to this, remaining free GO was only 8.18% at 0.5 hr of SGF and increased rapidly to 100% after the incubation with SIF with pancreatin for 6 hr. The low amount of remaining free GO from unencapsulated GO or free GO was very low in the first step since GO is water‐insoluble. Further incubation with pancreatin could solubilize the hydrophobic GO into the liquid. The sustained release effect by the encapsulation may be attributed to the strong hydrophobic interaction of GOZNs. Similar observation was also reported by Liu et al. (2019), pointing out that coating with a protein‐based delivery zein nanoparticles could control the release of the bioactive compound inside.

## 4 CONCLUSION

This preparation of ZNs demonstrated that the pH of the aqueous phase in the liquid‐liquid dispersion played an essential role in controlling their sizes. The increase of zein concentration and %GO loading led to a higher percentage of encapsulation efficiency and yield. FTIR and TEM revealed that the GO was encapsulated in the ZNs. Stable dispersion was obtained from the % zein concentration/ % GO loading which yielded GOZNs with size ranged 127.87–145.00 nm. The retention of GO in GOZNs was higher than those nonencapsulated GO during 60 day storage at both 4^o^C and −18^o^C. Zein coating has provided the control release of GO in simulated gastrointestinal fluid compared with the free GO.
